# Are scattered microsatellites weak chromosomal markers? Guided mapping reveals new insights into *Trachelyopterus* (Siluriformes: Auchenipteridae) diversity

**DOI:** 10.1371/journal.pone.0285388

**Published:** 2023-06-13

**Authors:** Chrystian Aparecido Grillo Haerter, Daniel Rodrigues Blanco, Josiane Baccarin Traldi, Eliana Feldberg, Vladimir Pavan Margarido, Roberto Laridondo Lui

**Affiliations:** 1 Instituto Nacional de Pesquisas da Amazônia, Manaus, Brasil; 2 Universidade Tecnológica Federal do Paraná, Campus Santa Helena, Paraná, Brasil; 3 Departamento de Genética, Instituto de Ciências Biológicas, Universidade Federal do Amazonas, Manaus, Brasil; 4 Universidade Estadual do Oeste do Paraná, Centro de Ciências Biológicas e da Saúde, Cascavel, Paraná, Brasil; Jeju National University, REPUBLIC OF KOREA

## Abstract

The scattered distribution pattern of microsatellites is a challenging problem in fish cytogenetics. This type of array hinders the identification of useful patterns and the comparison between species, often resulting in over-limited interpretations that only label it as "scattered" or "widely distributed". However, several studies have shown that the distribution pattern of microsatellites is non-random. Thus, here we tested whether a scattered microsatellite could have distinct distribution patterns on homeologous chromosomes of closely related species. The clustered sites of 18S and 5S rDNA, U2 snRNA and H3/H4 histone genes were used as a guide to compare the (GATA)_n_ microsatellite distribution pattern on the homeologous chromosomes of six *Trachelyopterus* species: *T*. *coriaceus* and *Trachelyopterus* aff. *galeatus* from the Araguaia River basin*; T*. *striatulus*, *T*. *galeatus* and *T*. *porosus* from the Amazonas River basin; and *Trachelyopterus* aff. *coriaceus* from the Paraguay River basin. Most species had similar patterns of the (GATA)_n_ microsatellite in the histone genes and 5S rDNA carriers. However, we have found a chromosomal polymorphism of the (GATA)_n_ sequence in the 18S rDNA carriers of *Trachelyopterus galeatus*, which is in Hard-Weinberg equilibrium and possibly originated through amplification events; and a chromosome polymorphism in *Trachelyopterus* aff. *galeatus*, which combined with an inversion polymorphism of the U2 snRNA in the same chromosome pair resulted in six possible cytotypes, which are in Hardy-Weinberg disequilibrium. Therefore, comparing the distribution pattern on homeologous chromosomes across the species, using gene clusters as a guide to identify it, seems to be an effective way to further the analysis of scattered microsatellites in fish cytogenetics.

## Introduction

Microsatellites, also known as short tandem repeats (STRs, [1]) or simple sequence repeats (SSRs, [2]), are stretches of DNA that consist of tandemly repeating di-, tri-, tetra- or penta-nucleotide motifs arranged through eukaryotic genomes [3,4] in both coding and noncoding regions [4,5]. Most microsatellites are located in the nucleus (nuSSR), although they can also be found in mitochondria (mtSSR) and chloroplasts (cpSSR) [4]. They are one of the most abundant and variable types of DNA sequences in the genome [3,5,6] and occur primarily due to slipped-strand mispairing and subsequent error(s) during DNA replication, repair or recombination [4,7]. However, the activity of transposable elements, mainly non-LTR retrotransposons, has also been reported as a major source of new microsatellites and their movement throughout the genome [4,8–11].

SSRs are recognized as powerful informative markers of genetic diversity and variability in both animal and plants [12–14]. In the last decades, they have been associated to several evolutionary and diversification aspects (see [3,10,15], including as potential causes of major structural chromosomal rearrangements, which seems to be facilitated by their high flexibility and low stability that creates fragile chromosomal sites [16,17]. In chromosomal mapping, although studies are still scarce for most species, they have provided a useful tool for understand the genome and chromosomal evolution of many different taxa [18–20]. They can be cytogenetically identified in large accumulations on a few chromosomal pairs [20,21] or with scattered signals throughout the chromosomes [20,22,23]. Furthermore, SSRs are commonly described associated to heterochromatic regions (e.g., [24,25]), often participating in its origin and increase (cHC) (for reviews, see [26–28]), and with crucial roles in the origin and evolution of specific chromosomes, mainly B chromosomes [22,23,29] and sex chromosomes [21,30,31]. However, in complement A chromosomes and populations without sex or B chromosomes, the cytogenetic studies are still concentrated only in the type of array and presence or absence of the marker, considering the whole karyotype [32,33].

One of the most challenging problems in expanding the use of microsatellites in fish cytogenetics is the scattered distribution pattern (e.g., [34–36], Table 1), because this type of array exhibit apparently random signals on the chromosomes. As a consequence, in most species without B or sex chromosomes, it hampers the description of useful distribution patterns to discuss evolutionary aspects as well as the comparison between karyotypes, often leading to oversimplified interpretations that simply label it as widely distributed or scattered. However, different lines of evidence indicated that the distribution of microsatellites is nonrandom [10]. They can influence in several aspects of the genome, including the nucleosome packing [37], methylation [38], high order chromatin structure [39–41] and splicing [42–44]. Microsatellites can also have enhancer functions [45–47], modulate gene expression [48–50] and participate of gene activity, recombination, DNA replication, cell cycle and mismatch repair (MMR) system [10,51]. Therefore, it is unlikely to believe that the scattered distribution pattern of SSRs in cytogenetic mappings is random and that it could not be helpful as chromosomal markers. In this scenario, considering that the scattered pattern consists in signals throughout the chromosomes, the association or colocalization with clustered sites in cytogenetic mapping, as already reported with several other types of DNA sequences [11,13,52], may provide an additional method to compare species and karyotypes. The clusters of previously mapped repetitive elements can be used as guide to identify homeologous chromosomes among species or populations, enabling comparative analyses beyond those with B or sex chromosomes.

**Table 1:** An overview of published cytogenetic mappings with microsatellites in neotropical fish species and their distribution patterns.

Specie	2n	Microsatellite/taxon	Distribution pattern	Main Contributions	Ref
**SILURIFORMES**					
Auchenipteridae					
*Ageneiosus inermis*	56	(GATA)_n_	Scattered	Karyoevolution	[32]
*Glanidium riberoi*	58	(GATA)_n_	Scattered	Karyoevolution	[53]
*Trachelyopterus galeatus*	58	(GATA)_n_	Scattered	B chromosomes	[22]
*Trachelyopterus* aff. *galeatus*	58	(GATA)_n_	Scattered	-	1
*Trachelyopterus coriaceus*	58	(GATA)_n_	Scattered	-	1
*Trachelyopterus* aff. *coriaceus*	58	(GATA)_n_	Scattered	B chromosomes	[23]
*Trachelyopterus striatulus*	58	(GATA)_n_	Scattered	B chromosomes	[23]
*Trachelyopterus porosus*	58	(GATA)_n_	Scattered	B chromosomes	[22]
Loricariidae					
*Ancistrus*	-	(GA)_15_ and (CA)_15_	Scattered	Sex chromosomes	[54]
*Astroblepus grixalvii*	52	(GA)_15_ and (CA)_15_	Subtelomeric	Karyoevolution	[55]
*Astroblepus homodon*	54	(GA)_15_ and (CA)_15_	Subtelomeric	Karyoevolution	[55]
*Harttia punctata*	58	(GATA)_n_	No signal	-	[56]
*Harttia duriventris*	55/56	(A)_30_, (CA)_15_ and (GA)_15_	Scattered/clustered	Sex chromosomes	[57]
*Harttia villasboas*	55/56	(A)_30_, (CA)_15_ and (GA)_15_	Scattered/clustered	Sex chromosomes	[57]
*Harttia rondoni*	54	(A)_30_, (CA)_15_ and (GA)_15_	Scattered/clustered	Sex chromosomes	[57]
*Hypostomus ancistroides*	68	(GATA)_n_	Scattered/dispersal	Karyoevolution	[33]
*Hypostomus iheringii*	80	(GATA)_n_	Scattered/dispersal	Karyoevolution	[33]
*Hypostomus nigromaculatus*	76	(GATA)_n_	Scattered/dispersal	Karyoevolution	[33]
*Hypostomus tapijara*	66	(GATA)_n_	Scattered/dispersal	Karyoevolution	[33]
*Panaqolus tankei*	52	(AC)_15_, (GA)_15_ and (GT)_15_	Scattered/subtelomeric	Karyoevolution	[58]
*Rinelocaria latirostris*	44	(GA)_15_	Dispersal/clustered	Karyoevolution	[59]
Heptapteridae					
*Imparfins schubarti*	58	(GA)_15_, (GACA)_4_, (GAA)_7_, (CAC)_5_, and (CA)_8_	Dispersal/clustered	Karyoevolution	[59,60]
*Imparfinis borodini*	50	(GA)_15_, (GACA)_4_, (GAA)_7_, (CAC)_5_, and (CA)_8_	Scattered/clustered	Karyoevolution	[60]
*Pimelodella cf*. *chagresi*		(CA)_15_ and (GA)_15_	Subtelomeric	Sex chromosomes	[61]
Pimelodidae					
*Bergiaria westermanni*	56	(GATA)_n_	Scattered	Sex chromosomes	[62]
*Steindachneridion scripta*	56	(GA)_15_	Dispersal/clustered	Karyoevolution	[59]
**GYMNOTIFORMES**					
Gymnotidae					
*Gymnotus sylvius*	40	(A)_30_, (C)_30_, (CA)_15_, (GA)_15_, (GC)_15_, (TTA)_10_, (CAA)_10_, and (GAG)_10_	Scattered, clustered, and mixed	Karyoevolution	[63]
*Gymnotus cuia*	54	(A)_30_, (C)_30_, (CA)_15_, (GA)_15_, (GC)_15_, (TTA)_10_, (CAA)_10_, and (GAG)_10_	Scattered, clustered, and mixed	Karyoevolution	[63]
*Gymnotus pantanal*	39/40	(A)_30_, (C)_30_, (CA)_15_, (GA)_15_, (GC)_15_, (TTA)_10_, (CAA)_10_, and (GAG)_10_	Scattered, clustered, and mixed	Karyoevolution	[63]
*Gymnotus capanema*	34	(A)_30_, (C)_30_, (CA)_15_, (GA)_15_, (GC)_15_, (TTA)_10_, (CAA)_10_, and (GAG)_10_	Scattered, clustered, and mixed	Karyoevolution	[63]
*Gymnotus carapo*	42	(A)_30_, (C)_30_, (CA)_15_, (GA)_15_, (GC)_15_, (TTA)_10_, (CAA)_10_, and (GAG)_10_	Scattered, clustered, and mixed	Karyoevolution	[63]
**GOBIIFORMES**					
Gobiidae					
*Gobionellus oceanicus*	56	(CA)_15_	Subtelomeric/dispersal	Sex chromosomes	[64]
*Gobionellus stomatus*	56	(CA)_15_	Subtelomeric/dispersal	Sex chromosomes	[64]
**CHARACIFORMES**					
Anostomidae					
*Leporinus elongatus*	54	(A)_30_, (C)_30_, (CA)_15_, (GC)_15_, (GA)_15,_ (GAA)_10_, (CAG)_10_, and (CAT)_10_	Scattered, clustered, and mixed	Sex chromosomes	[65]
*Leporinus conirostris*	54	(A)_30_, (C)_30_, (CA)_15_, (GC)_15_, (GA)_15,_ (GAA)_10_, (CAG)_10_, and (CAT)_10_	Scattered, clustered, and mixed	Sex chromosomes	[65]
*Leporinus obtusidens*	54	(A)_30_, (C)_30_, (CA)_15_, (GC)_15_, (GA)_15,_ (GAA)_10_, (CAG)_10_, and (CAT)_10_	Scattered, clustered, and mixed	Sex chromosomes	[65]
*Leporinus reinhardti*	54	(A)_30_, (C)_30_, (CA)_15_, (GC)_15_, (GA)_15,_ (GAA)_10_, (CAG)_10_, and (CAT)_10_	Scattered, clustered, and mixed	Sex chromosomes	[65]
*Astyanax altiparanae*	50	(CA)_15_, (GA)_15_, (CG)_15_, (GACA)_4_ and (GATA)_8_	Scattered, clustered, and mixed	B chromosome	[18]
*Astyanax fasciatus*	46	(CA)_15_, (GA)_15_, (CG)_15_, (GACA)_4_ and (GATA)_8_	Scattered, clustered, and mixed	B chromosome	[18]
*Astyanax marionae*	48	(CA)_15_, (GA)_15_, (CG)_15_, (GACA)_4_ and (GATA)_8_	Scattered, clustered, and mixed	B chromosome	[18]
*Astyanax*. *schubarti*	36	(CA)_15_, (GA)_15_, (CG)_15_, (GACA)_4_ and (GATA)_8_	Scattered, clustered, and mixed	B chromosome	[18]
*Astyanax mexicanus*	50	(CA)_15_, (GA)_15_, (CG)_15_, (GACA)_4_ and (GATA)_8_	Scattered, clustered, and mixed	B chromosome	[18]
		Lebiasinidae			
*Pyrrhulina australis*	40	(CA)_15_, (GA)_15_	Scattered, clustered, and mixed	Sex chromosomes	[66,67]
*Pyrrhulina aff*. *australis*	40	(CA)_15_, (GA)_15_	Scattered, clustered, and mixed	Sex chromosomes	[66,67]
*Pyrrhulina brevis*	42	(CA)_15_, (GA)_15_	Scattered, clustered, and mixed	Sex chromosomes	[66]
*Pyrrhulina semifasciata*	41	(CA)_15_, (GA)_15_	Scattered, clustered, and mixed	Sex chromosomes	[66]
*Lebiasina bimaculata*	36	(CA)_15_, (GA)_15_, (CAT)_10_, and (CGG)_10_	Scattered, clustered, and mixed	Sex chromosomes	[68]
*Lebiasina melanoguttata*	36	(CA)_15_, (GA)_15_, (CAT)_10_, and (CGG)_10_	Scattered, clustered, and mixed	Sex chromosomes	[68]
		Erythrinidae			
*Erythrinus erythrinus*	51	(CA)_15_, (CAA)_10_, (CAC)_10_, (CAG)_10_, (CAT)_10_, (CGG)_10_, (GA)_15_, (GAA)_10_, (GAG)_10_ and (TA)_15_	Scattered, clustered, and mixed	Sex chromosomes	[69,70]
*Hoplias intermedius*	50	(CAA)_10_, (GA)_15_ and (CA)_15_	Scattered, clustered, and mixed	Karyoevolution	[71]
*Hoplias brasiliensis*	50	(A)_30,_ (CA)_15_, (CAA)_10_, (GA)_15,_ (CA)_15,_ and (CAC)_10_	Scattered, clustered, and mixed	Karyoevolution	[71,72]
*Hoplias aimara*	50	(A)_30,_ (CA)_15_, (CAA)_10_, (GA)_15,_ (CA)_15,_ and (CAC)_10_	Scattered, clustered, and mixed	Karyoevolution	[71,72]
*Hoplias lacerdae*	50	(A)_30,_ (CA)_15_, (CAA)_10_, (GA)_15,_ (CA)_15,_ and (CAC)_10_	Scattered, clustered, and mixed	Karyoevolution	[71,72]
*Hoplias malabaricus*	50	(A)_30_, (C)_30_, (CA)_15_, (GA)_15_, (TA)_15_, (CAC)_10_, (CAT)_10_, (GAC)_10_,(GAG)_10_, and (CGG)_10,_ (GC)_15_, (CAA)_10_, (CAC)_10_, (CAT)_10_, (TAA)_10_	Scattered, clustered, and mixed	Sex chromosomes	[73–75]
*Hoplias australis*	50	(A)_30_, (CA)_15_, (GA)_15_, and (CAC)_10_	Scattered, clustered, and mixed	Karyoevolution	[72]
*Hoplias curupira*	50	A)_30_, (CA)_15_, (GA)_15_, and (CAC)_10_	Scattered, clustered, and mixed	Karyoevolution	[72]
*Hoplias intermedius*	50	A)_30_, (CA)_15_, (GA)_15_, and (CAC)_10_	Scattered, clustered, and mixed	Karyoevolution	[72]
		Crenuchidae			
*Characidium zebra*	50	(A)_30_, (C)_30_, (CA)_15_, (CAA)_10_, (CAC)_10_, (CAG)_10_, (CAT)_10_, (CG)_15_, (CGG)_10_, (GA)_15_, (GAA)_10_, (GAC)_10_, (GACA)_4_, (GAG)_10_, (TA)_15_, (TAA)_10_ and (GATA)_7_	Scattered, clustered, and mixed	Karyoevolution	[76]
*Characidium gomesi*	50	A)_30_, (C)_30_, (CA)_15_, (CAA)_10_, (CAC)_10_, (CAG)_10_, (CAT)_10_, (CG)_15_, (CGG)_10_, (GA)_15_, (GAA)_10_, (GAC)_10_, (GACA)_4_, (GAG)_10_, (TA)_15_, (TAA)_10_ and (GATA)_7_	Scattered, clustered, and mixed	Sex chromosomes	[76,77]
*Hepsetus odoe*	58	(A)_30_, (CA)_15_, (GA)_15_, (CAC)_10_, (CGG)_10_, (GAA)_10_ and (GAG)_10_	Scattered, clustered, and mixed	Karyoevolution	[78]
*Characidium cf*. *zebra*	50	(CA)_15_, (GA)_15_, (CG)_15_, and (TTA)_10_	Subtelomeric/clustered	Sex chromosomes	[77]
*Characidium vidali*	50	CA)_15_, (GA)_15_, (CG)_15_, and (TTA)_10_	Subtelomeric/clustered	Sex chromosomes	[77]
*Characidium pterostictum*	50	CA)_15_, (GA)_15_, (CG)_15_, and (TTA)_10_	Subtelomeric/clustered	Sex chromosomes	[77]
*Characidium timbuiense*	50	CA)_15_, (GA)_15_, (CG)_15_, and (TTA)_10_	Subtelomeric/clustered	Sex chromosomes	[77]
*Characidium lanei*	50	CA)_15_, (GA)_15_, (CG)_15_, and (TTA)_10_	Subtelomeric/clustered	Sex chromosomes	[77]
*Characidium alipioi*	50	(CA)_15_, (GA)_15_ and (GAG)_10_	Scattered/clustered	B chromosomes	[79]
		Anostomidae			
*Hypomasticus copelandii*	54	(CA)_15_, (GA)_15_	Subtelomeric	Karyoevolution	[80]
*Hypomasticus steindachneri*	54	(CA)_15_, (GA)_15_	Subtelomeric	Karyoevolution	[80]
		Characidae			
*Astyanax scabripinnis*	50	(CA)_15_, (CAC)_10_, (CAG)_10_, (CAT)_10_, (GA)_15_, (GAA)_10_, (GAG)_10_, (GC)_15_ and (GATA)_n_	Scattered, clustered, and mixed	Karyoevolution	[30]
*Astyanax bockmanni*	-	(AG)_15_	Abundant in B chromosomes	B chromosomes	[81]
*Astyanax fasciatus*	45–47	(AG)_15_	Abundant in B chromosomes	B chromosomes	[81]
*Astyanax paranae*	-	(AG)_15_	Abundant in B chromosomes	B chromosomes	[81]
*Triportheus trifurcatus*	52	(CA)_15_, (CAA)_10_, (CAC)_10_, (CAG)_10_, (CAT)_10_, (CGG)_10_, (GA)_15_, (GAA)_10_ and (TA)_15_	Scattered, clustered, and mixed	Sex chromosomes	[82]
*Hyphessobrycon eques*	52	(A)_30_, (CA)_15_, (CAG)_10_ and (GATA)_8_	Scattered, clustered, and mixed	Karyoevolution	[83]
		Parodontidae			
*Apareiodon* sp.	54	(GATA)_n_	Scattered, clustered, and mixed	Sex chromosomes	[75,84]
*Apareiodon machrisi*	54	(GATA)_n_	Scattered, clustered, and mixed	Karyoevolution	[85]
*Apareiodon cavalcante*	54	(GATA)_n_	Scattered, clustered, and mixed	Karyoevolution	[85]
*Apareiodon* sp. 1	54	(GATA)_n_	Scattered, clustered, and mixed	Karyoevolution	[85]
*Apareiodon* sp. 2	54	(GATA)_n_	Scattered, clustered, and mixed	Karyoevolution	[85]
		Prochilodontidae			
*Semaprochilodus insignis*	54	(CA)_22_, (GA)_20_, (GT)_17_, (CT)_26_, (CT)_14_GT(CT)_5_(CG)_2_(CT)_9_, (GT)_9_CA(GT)_7_CG(GT)_19_	Dispersal/clustered	Sex chromosomes	[70]
*Semaprochilodus taeniurus*	54	CA)_22_, (GA)_20_, (GT)_17_, (CT)_26_, (CT)_14_GT(CT)_5_(CG)_2_(CT)_9_, (GT)_9_CA(GT)_7_CG(GT)_19_	Dispersal/clustered	Sex chromosomes	[70]

Auchenipteridae, known as the driftwood catfishes, is one of the 43 Siluriformes families. They are restricted but widely distributed in South America and have 25 genera and 128 valid species [86]. Among them, *Trachelyopterus* (with 17 valid species) is one of the most studied genera owing to high morphological similarity and, hence, a controversial taxonomic history [87]. The physical localization of 18S rDNA, 5S rDNA, H3/H4 histone genes and snRNA U2 is well documented in six species: *T*. *coriaceus*, *T*. *striatulus*, *Trachelyopterus* aff. *galeatus* (cited as *Parauchenipterus galeatus*) [88–90], *T*. *porosus* [90,91], *T*. *galeatus* (cited as *P*. *galeatus*), *Trachelyopterus* aff. *coriaceus* (cited as *Trachelyopterus* sp.) [23,90]; whereas the SSR (GATA)_n_ sites were described in only three species: *T*. *galeatus* [22,23], *T*. *porosus* [22] and *Trachelyopterus* aff. *coriaceus* (cited as *Trachelyopterus* sp.) [23]. In B chromosomes, the (GATA)_n_ sequence has a particularly clustered pattern, and provided valuable data about the origin and evolution of these extra chromosomes in Auchenipteridae. On the other hand, in complement A chromosomes it presented a scattered distribution pattern and was not able to reveal diversity or specific traits [22,23]. Therefore, these *Trachelyopterus* species constitutes an excellent model to be investigated through the integrated mapping, since it has (1) scattered signal of a microsatellite in all complement A chromosomes; (2) well known clusters of 18S rDNA, 5S rDNA, H3/H4 histone genes and U2 snRNA and; (3) they are closely related species with high similar karyotypes, which could test the integrated mapping efficiency.

In this paper the microsatellite (GATA)_n_ distribution pattern was compared among the carriers of the 5S and 18S rDNA, H3/H4 histone genes and U2 snRNA sites, in six *Trachelyopterus* species. The clustered repetitive elements sites were used as a guide to specific chromosomal pairs. We intended to test whether a widely distributed microsatellite could have distinct distribution patterns on homeologous chromosomes among closely related species, revealing unknown chromosome arrangements as well as architecture interaction between them.

## Materials and methods

### Ethics statement

Fish collections were authorized by Instituto Chico Mendes de Conservação da Biodiversidade (ICMBio, Permit number 49379), and the experimental procedures were approved by the Ethics Committee on Animal Experimentation and Practical Classes at Unioeste (09/13-CEEAAP/Unioeste). The use of the genetic data in this study was authorized by the National System for the Management of Genetic Heritage and Associated Traditional Knowledge (SisGen n° AFFBCC7).

### Specimens and DNA extraction

Four *Trachelyopterus* species, including two possible new ones, were analyzed: *Trachelyopterus galeatus* Linnaeus, 1766 (04 males and 07 females) from Catalão lake, Manaus, Amazon River basin, 03°09’47’’S; 59°54’2’’W (Instituto Nacional de Pesquisas na Amazônia—INPA 57939); *Trachelyopterus coriaceus* Valenciennes, 1840 (04 males and 03 females) from Araguaia River, São Miguel do Araguaia, 13°08’52,7’’S; 50°25’02,8’’W (Museu da Universidade de São Paulo—MZUSP 106766); *Trachelyopterus porosus* Eigenmann and Eigenmann, 1888 (04 males and 04 females) from Catalão lake, Manaus, Amazon River basin, 03°09’47’’S; 59°54’29"W (Instituto Nacional de Pesquisas da Amazônia—INPA 57940); *Trachelyopterus* aff. *galeatus* (06 males and 10 females) from Araguaia River (suggested as new species), São Miguel do Araguaia, 13°08’52,7’’S; 50°25’02,8’W (MZUSP 110803); *Trachelyopterus striatulus* Steindachner, 1877 (03 males and 03 females) from Verde lagoon, Doce River basin, Marliéria, 19°49’44,5’’S; 42°37’52.5’’W (MZUSP 109798); and *Trachelyopterus* aff. *coriaceus* (02 males and 01 female) from Arrombado lagoon (suggested as new species), Bento Gomes River basin, Poconé, 16°25’40,9’’S; 56°25’07,4’’W (MZUSP 110806). Genomic DNA was extracted from the liver of all species according to Sambrook et al. (2001).

### Probes labeling and sequencing

The 18S rDNA and 5S rDNA probes were obtained through polymerase chain reaction—PCR [90] using the primers NS1 and NS8 [92], 5SA and 5SB [93]. The 18S rDNA probes were labeled with Biotin-16-dUTP (Bio-Nick-Translation, Roche) according to manufacturer’s instructions and detected with Antibiotin-Avidin-FITC / Antiavidin-Biotin (Roche). The 5S rDNA were labeled through PCR with Tetramethyl-Rhodamine-5-dUTP (Roche). The H3 and H4 histone genes fragments were amplified from *Trachelyopterus galeatus* genome using the primers H3F and H3R [94]; and H4F2s and H4F2er [95]. Both probes were labeled through PCR with Tetramethyl-Rhodamine-5-dUTP (Roche) [90]. The U2 snRNA fragments were amplified from *Trachelyopterus galeatus* genome with the primers U2F and U2R [96]. The U2 snRNA probes were labeled through PCR with Fluorescein-12-dUTP (Roche) [90]. The (GATA)_n_ probes were generated through PCR in the absence of a template, using only the primers [97] and labeled with Tetramethyl-Rhodamine-5-dUTP (Roche).

All PCR products were sequenced in both ways, forward and reverse, using the ABI 3730 DNA Analyzer with the BigDye Terminator v3.1 Cycle Sequencing Kit (code 4337456) and the Sequencing Analysis software 5.3.1. The consensus sequence was generated by the Bioedit Sequence Alignment Editor [98]. The fragments identity was confirmed through BLASTn 2.11.0 (National Center for Biotechnology Information) [99].

### Cytogenetic analyses and Fluorescent in situ hybridization

The samples were treated with a 0.02% colchicine solution (1 ml/100g of body weight) for 30–40 min and sequentially euthanized by clove oil overdose [100] (according to the ethics committee on animal experimentation and practical classes at Unioeste: 09/13—CEEAAP / Unioeste). The mitotic chromosomes were obtained from anterior kidney cells [101]. The chromosome morphology was classified according to [102].

Fluorescent in situ hybridization (FISH) was carried out with 77% of stringency [103] with some suggested modifications [104]. Clusters of 18S rDNA, 5S rDNA, snRNA U2, H3 and H4 histone genes were used as guide to integrative (guided) mapping with the SSR (GATA)_n_. The digital images were captured by the DP Controller 3.2.1.276 software using an Olympus DP71 digital camera connected to the BX61 epifluorescence microscope (Olympus America Inc., Center Valley, PA, United States of America). The Hardy–Weinberg equilibrium (HWE) and Chi-squared test were performed using the Hardy-Weinberg (HW) testing program [105].

## Results

### DNA sequencing analysis

The U2 snRNA and both H3 and H4 histone genes were sequenced with forward and reverse primers. The U2 snRNA had a 199-bp consensus sequence (Genbank ID: OK166555.1) with 96,4% similarity with *Parabotia fasciatus* U2 snRNA gene (Genbank ID: MG874999.1). The histone gene H3 had a 448-bp consensus sequence (Genbank ID: OK166556.1) with 91.57% similarity with the *Pimelodus microstoma* H3 histone gene (Genbank ID: MT094432.1). The histone gene H4 had a 269-bp consensus sequence (Genbank ID: OK166557.1) with 90.09% similarity with the *Pimelodus microstoma* H4 histone gene (Genbank ID: MT094433.1).

### Cytogenetic analysis

#### *Trachelyopterus* aff. *galeatus*–Araguaia River basin

**Fig 1:**
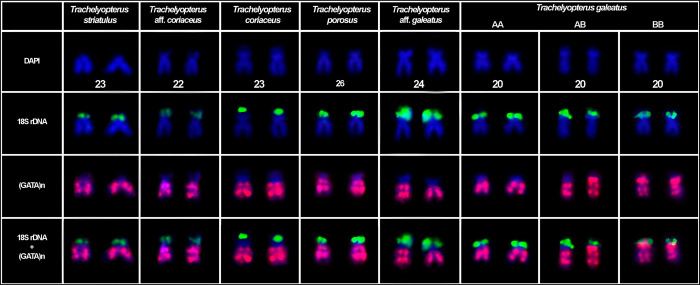
SSR (GATA)n distribution pattern in the 18S rDNA carrier of *Trachelyopterus* species. The 18S rDNA probes are in green signal and the (GATA)n probes in red signal.

**Fig 2:**
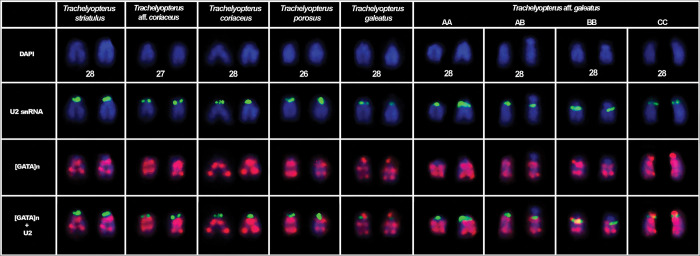
SSR (GATA)n distribution pattern in the U2 snRNA carrier of *Trachelyopterus* species. U2 snRNA probes in green signal and (GATA)n probes in red signal. A1A1: Both chromosomes with U2 snRNA sites in the short arm and SSR (GATA)n only in the long arm; A1A2: One chromosome with U2 snRNA site in the long arm and another chromosome with the U2 snRNA site in the short arm, both with the SSR (GATA)n only in the long arm of the chromosome; A2A2: Both chromosomes with U2 snRNA sites in the short arm and the SSR (GATA)n only in the long arm; A3A3: Both chromosomes with U2 snRNA sites in the short arm of the chromosome and the SSR (GATA)n in the long and short arm of the chromosome.

**Fig 3:**
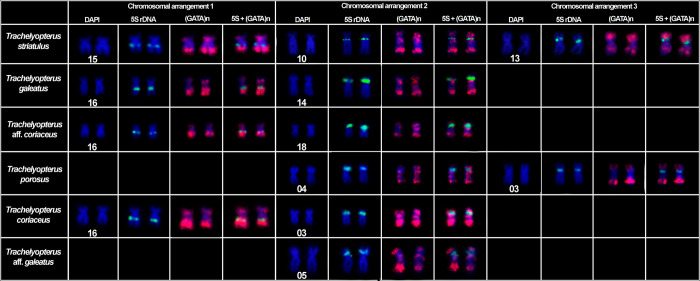
SSR (GATA)n distribution pattern in the 5S rDNA carrier of *Trachelyopterus* species. The 5S rDNA probes are in green signal and the (GATA)n probes in red signal.

**Fig 4:**
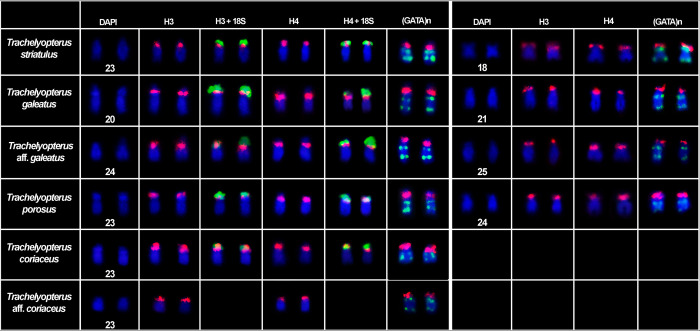
SSR (GATA)n distribution pattern in the H3 and H4 histone genes carrier of *Trachelyopterus* species. The H3/H4 histone genes probes are in red signal, The 18S rDNA and (GATA)n probes in green signal.

**Fig 5:**
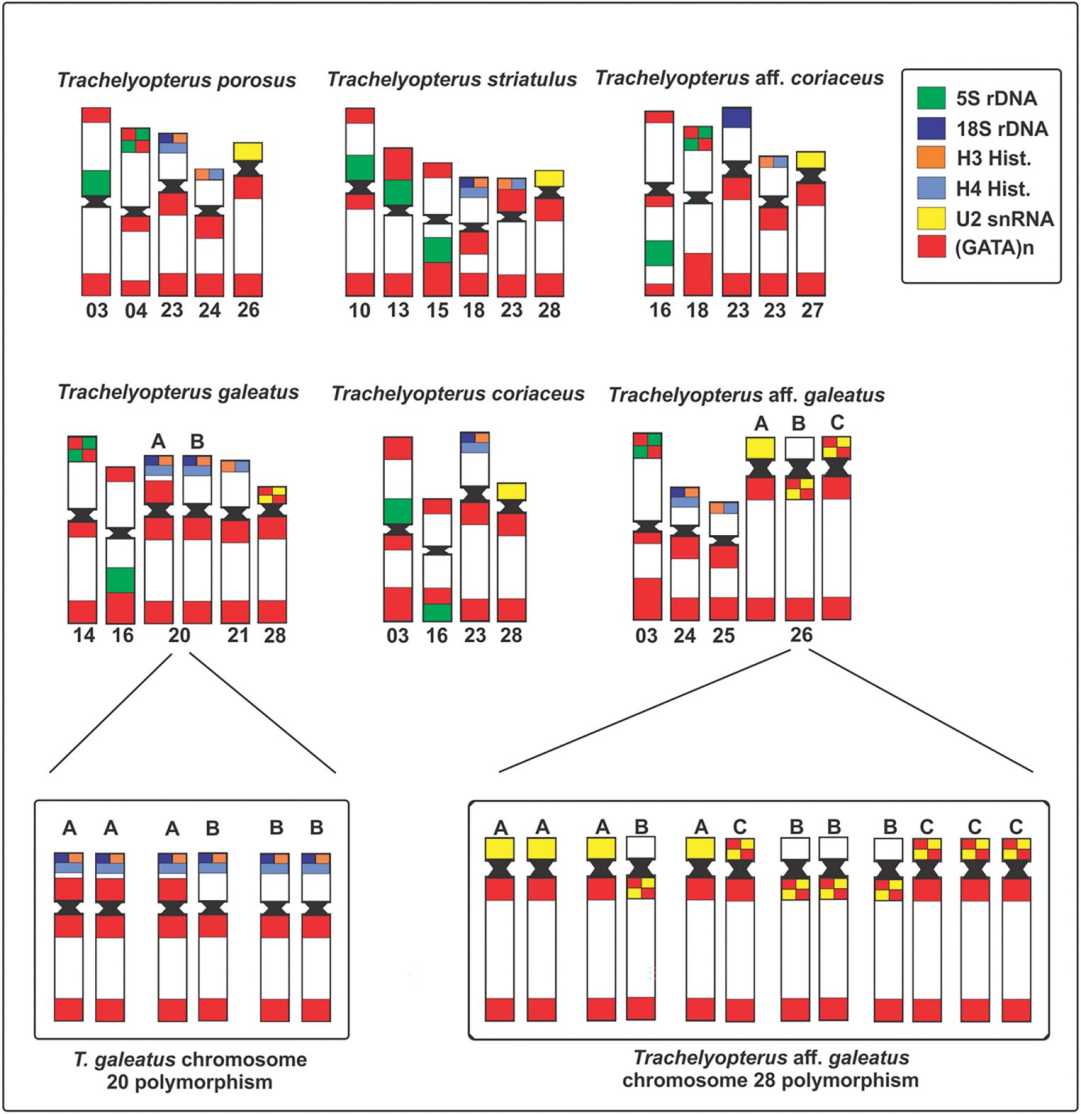
Idiogram of the (GATA)_n_ distribution pattern in the chromosomes carrying the 5S rDNA, 18S rDNA, H3 and H4 histone genes and U2 snRNA.

This species had 2n = 58 chromosomes for both sexes (7 males and 9 females). The microsatellite (GATA)_n_ were found spread throughout the chromosomes. The chromosomal pair 24, which bears the 18S rDNA, H3 and H4 histone genes loci, exhibited two blocks of the SSR (GATA)_n_ in the terminal position of the long arm and none in the short arm (Figs 1 and 4). The chromosomal pair 25, which bears another H3 and H4 histone genes loci, also had two blocks of the SSR (GATA)_n_ in the long arm and none in the short arm (Fig 4). The chromosomal pair 26, which bear the U2 snRNA locus, exhibited a chromosome polymorphism associated to the SSR (GATA)_n_ distribution pattern (Fig 2), which combined with the U2 snRNA distribution pattern evidenced three chromosomal forms, including a new one, here referred as C chromosomal form: (A) U2 snRNA sites in the short arm with SSR (GATA)_n_ blocks only in the long arm of the chromosome; (B) U2 snRNA sites in the long arm with SSR (GATA)_n_ blocks only in the long arm of the chromosome; and (C) U2 snRNA sites in the short arm with SSR (GATA)_n_ blocks in both the short and long arm of the chromosome. From all chromosomal forms, six combinations could be hypothesized and four of them were found in the sample: AA (1 female), AB (2 males and 3 females), AC, BB (1 male and 5 females), BC, CC (2 males and 2 females) (Figs 2 and 5, Table 2). The χ2 value for Hardy-Weinberg equilibrium (HWE) was 15.99 (Df = 03; p = <0.05). The chromosomal pair 3 (5S rDNA) had three blocks of the SSR (GATA)_n_, two in the short arm (one in the terminal position and one in interstitial position) and one in the terminal position of the long arm (Fig 3).

**Table 2:** Hardy-Weinberg Equilibrium test for *Trachelyopterus galeatus* (GATA)n polymorphism from Amazonas River basin and *Trachelyopterus* aff. *galeatus* U2 snRNA and (GATA)n polymorphism from Araguaia River basin.

Genotype	Genotype Absolut frequencies		Genotype relative frequencies		χ2	p value*
*Trachelyopterus galeatus* (GATA)_n_ polymorphism—Amazonas						
	**observed**	**expected**	**observed**	**expected**		
**AA**	3 (2♂ 1♀)	3.841	0.273	0.349	0.184	
**AB**	7(2♂ 5♀)	5.318	0.636	0.483	0.531	
**BB**	1(1♂)	1.841	0.091	0.167	0.384	
				**Total**	**1.099**	**0.2942**
*Trachelyopterus* aff. *galeatus* U2 snRNA and (GATA)_n_ polymorphism—Araguaia						
	**observed**	**expected**	**observed**	**expected**		
**AA**	1 (1♀)	0.766	0.063	0.048	0.071	
**AB**	5 (2♂ 3♀)	3.719	0.313	0.232	0.441	
**AC**	0	1.75	0	0.109	1.75	
**BB**	6 (1♂ 5♀)	4.516	0.375	0.282	0.487	
**BC**	0	4.25	0	0.266	4.25	
**CC**	4 (2♂ 2♀)	1	0.25	0.063	9	
				**Total**	**15.999**	**0.0011**

#### *Trachelyopterus galeatus*–Amazon River basin

This species had 2n = 58 chromosomes for both sexes (7 males and 9 females). The chromosomal pair 20, which bears the 18S rDNA, H3 and H4 histone genes loci, has shown a chromosome polymorphism (Fig 1). It consists in two different organizations of the SSR (GATA)_n_ in the chromosome: two SSR (GATA)_n_ blocks in the terminal and interstitial position of long arm of the chromosome (A form); or two SSR (GATA)_n_ blocks in the terminal position and interstitial position of the long arm and one in the interstitial position of the short arm of the chromosome (B form). Three combinations of these polymorphic forms were found in this population: (AA) both chromosomes with two SSR (GATA)_n_ blocks in the terminal and proximal position of long arm of the chromosome (2 males and 1 female); (AB) one chromosome with two SSR (GATA)_n_ blocks in the terminal and proximal position of long arm of the chromosome and another chromosome with two SSR (GATA)_n_ blocks in the terminal and proximal position of the long arm of the chromosome and one in the proximal position of the short arm of the chromosome (2 males and 5 females); (BB) both chromosomes with two SSR (GATA)_n_ blocks in the terminal and proximal position of the long arm of the chromosome and one in the proximal position of the short arm of the chromosome (1 male). The chromosomal pair 28, bearing the U2 snRNA locus, had two blocks of the SSR (GATA)_n_ in the long arm and one in terminal position of the short arm. The chromosome polymorphism was found in Hardy-Weinberg equilibrium (HWE) (χ2 = 1.099; Df = 01; p = <0.05). The chromosomal pair 24, also bearing a H3 and H4 histone genes loci, had two blocks of the SSR (GATA)_n_ in the long arm and none in the short arm (Fig 2). The chromosomal pair 14, bearing the first 5S rDNA locus, exhibited three blocks of the SSR (GATA)_n_, two in the short arm (one in the terminal position and one in interstitial position) and one in the terminal position of the long arm (Fig 4). The chromosomal pair 16, bearing the second 5S rDNA locus, had two blocks of the SSR (GATA)_n_, one in the terminal position of the short and another in the long arm (Fig 3).

***Trachelyopterus porosus*–Amazon River basin.** This species had 2n = 58 chromosomes for both sexes (4 males and 4 females). The microsatellite (GATA)_n_ were found spread throughout the chromosomes. The chromosomal pair 23, which bears the 18S rDNA, H3 and H4 histone genes loci, had two blocks of the SSR (GATA)_n_ in the long arm and none in the short arm (Figs 1 and 4). The chromosomal pair 24, also bearing a H3 and H4 histone genes loci, had two blocks of the SSR (GATA)_n_ in the long arm and none in the short arm (Fig 4). The chromosomal pair 26, which bears the U2 snRNA locus, exhibited two blocks of the SSR (GATA)_n_ in the long arm and none in the short arm (Fig 2). The chromosomal pair 3, which bears the first 5S rDNA locus, exhibited had two blocks of the SSR (GATA)_n_, one in the terminal position of the short and another in the long arm. The chromosomal pair 4, bearing the second 5S rDNA locus, had three blocks of the SSR (GATA)_n_, two in the short arm (one in the terminal position and one in interstitial position) and one in the terminal position of the long arm (Fig 3).

***Trachelyopterus coriaceus*–Araguaia River basin.** This species had 2n = 58 chromosomes for both sexes (4 males and 3 females). The microsatellite (GATA)_n_ were found spread throughout the chromosomes. The chromosomal pair 23, which bears the 18S rDNA, H3 and H4 histone genes loci, exhibited two blocks of the SSR (GATA)_n_ in the long arm and none in the short arm (Figs 1 and 4). The chromosomal pair 28, bearing the U2 snRNA loci, had two blocks of the SSR (GATA)_n_ in the long arm and none in the short arm (Fig 2). The chromosomal pair 3, which bears the first 5S rDNA locus, had three blocks of the SSR (GATA)_n_, two in the short arm (one in the terminal position and one in interstitial position) and one in the terminal position of the long arm. The chromosomal pair 16, bearing the second 5S rDNA locus, had two blocks of the SSR (GATA)_n_, one in the terminal position of the short and another in the long arm (Fig 3).

#### *Trachelyopterus* aff. *coriaceus*–Paraguay river basin

This species had 2n = 58 chromosomes for both sexes (2 males and 1 female). The microsatellite (GATA)_n_ were found spread throughout the chromosomes. The chromosomal pair 22, bearing the 18S rDNA locus, had two blocks of the SSR (GATA)_n_ in the long arm and none in the short arm (Fig 1). The chromosomal pair 23, which bears the H3 and H4 histone genes loci, exhibited two blocks of the SSR (GATA)_n_ in the long arm and none in the short arm (Fig 4). The chromosomal pair 27 bearing the U2 snRNA locus, exhibited two blocks of the SSR (GATA)_n_ in the long arm and none in the short arm (Fig 2). The chromosomal pair 16, bearing the first 5S rDNA locus, had two blocks of the SSR (GATA)_n_, one in the terminal position of the short and another in the long arm. The chromosomal pair 18, which bears the second 5S rDNA locus, had three blocks of the SSR (GATA)_n_, two in the short arm (one in the terminal position and one in interstitial position) and one in the terminal position of the long arm (Fig 3).

#### *Trachelyopterus striatulus*–Doce River basin

This species had 2n = 58 chromosomes for both sexes (3 males and 3 females). The microsatellite (GATA)_n_ were found spread throughout the chromosomes. The chromosomal pair 23, which bears the 18S rDNA, H3 and H4 histone genes loci, exhibited two blocks of the SSR (GATA)_n_ in the long arm and none in the short arm (Figs 1 and 4). The chromosomal pair 18, also bearing a H3 and H4 histone genes loci, had had two blocks of the SSR (GATA)_n_; however, one in terminal position of the long arm and another in the interstitial position of the short arm (Fig 4). The chromosomal pair 28, which bears the U2 snRNA locus, exhibited two blocks of the SSR (GATA)_n_ in the long arm and none in the short arm (Fig 2). The chromosomal pair 10, bearing the first 5S rDNA locus, had three blocks of the SSR (GATA)_n_, two in the long arm (one in the terminal position and one in interstitial position) and one in the terminal position of the short arm. The chromosomal pair 13 and 15, which bears another two 5S rDNA locus, exhibited two blocks of the SSR (GATA)_n_, one in the terminal position of the short and another in the long arm (Fig 3).

## Discussion

The (GATA)_n_ repeats, molecular components of the Bkm satellite DNA (Banded krait minor-satellite DNA), are widely distributed in the genome of higher organisms [106]. As expected, in all *Trachelyopterus* species of this study it was found scattered throughout the chromosomes (summarized in Fig 5). This type of array and has been described to different SSRs and taxa, such as plants [107,108], amphibians [20], fungus [109] and fish [22,23,33]. In Auchenipteridae, it was already reported in *T*. *porosus and T*. *galeatus* [22], *Trachelyopterus* aff. *coriaceus* (cited as *Trachelyopterus* sp. [23], and now it can also be seen in *T*. *coriaceus*, *T*. *striatulus* and *Trachelyopterus* aff. *galeatus*. This type of scattered array could be explained by the activity of transposable elements, which are a major source of new microsatellites and can also drive it throughout the genome [8–11] and/or to chromosomal rearrangements, as already proposed for a close genus, *Hypostomus* [33]. Though the (GATA)_n_ repeats have been associated to sex chromosome in different organisms [21,110–112], no differences between males and females have been found in these populations.

In the integrated mapping with the 18S rDNA, the chromosomal pair of all species had almost the same SSR (GATA)_n_ distribution pattern, characterized by the absence of the microsatellite only in the short arm (Fig 1). It was also reported in a close species, *Glanidium ribeiroi* [53], but it was unexplored yet. Usually, SSRs can constitute a larger fraction of noncoding DNA, but are rare in protein-coding regions [10] mainly due to negative selection against frameshift mutations in coding regions, as evidenced in plants, primates, and microorganisms [113]. Comparative studies showed that only repeats in multiples of three may develop evenly in both regions [5,113], since RNA bases are read as triplets and other types could result in frameshift mutations [113,114]. In this way, the absence of the (GATA)_n_ sequence in the short arm of the 18S rDNA chromosomes, might suggest a negative impact of the microsatellite near the coding areas of these species.

However, this hypothesis is contradicted by some evidences: (a) *T*. *galeatus* from the Amazon River basin had a (GATA)_n_ block in the short arm of the 18S rDNA carrier; (b) there is overlaid signal between the (GATA)_n_ sequence and the 5S rDNA and U2 snRNA sites; and (c) the (GATA)_n_ sequence is distributed throughout the chromosomes, indicating that it could also be near other unmapped gene sequences. Therefore, the non-existence of (GATA)_n_ signal in the short arm might only be related to a spatial issue, since besides the 18S rDNA, the short arm of most species also carries the H3 and H4 histone genes and, consequently, there could not be enough space for large amounts of (GATA)_n_ sequence detectable through Fluorescent *in situ* Hybridization, which needs targets of at least 1kb to express significative results [115].

In contrast to the other species of our sample, *T*. *galeatus* from the Amazon River basin was the only one to possess the (GATA)_n_ sequence in the short arm of the 18S rDNA carrier. However, it was identified in a polymorphic state, which appears to be neutral, since the heterozygous has no different adaptive value compared to other forms (Table 2). Although other mechanisms have been proposed to explain how microsatellites can arise and expand over time, e.g., double-stranded DNA recombination (unequal crossing over and gene conversion), mismatch/double-strand break repair, and retrotransposition; replication slippage is still considered one of the main mechanisms thus far [3,4,116]. It also seems to be the most parsimonious to explain the origin of the microsatellite (GATA)_n_ in the short arm of the 18S rDNA carrier in *T*. *galeatus* from the Amazon River basin. In this case, a small number of repeats (proto-microsatellite) is required before DNA polymerase slippage can extend the number of repeats, originating a new microsatellite [114], which seems not to be a problem in the genome of these *Trachelyopterus* species, as it is highly enriched with the (GATA)_n_ repeats. Once generated, the new microsatellite can undergo several reduction or expansion events over time (see [3]), which can lead to the formation of large microsatellite blocks, such as the one visualized in *T*. *galeatus* from the Amazon River basin. On the other hand, the polymorphic state observed in this population may have arisen from a crossing between individuals possessing the new trait and those with the original condition.

The (GATA)_n_ distribution pattern in the U2 snRNA chromosomes was conserved among most species. However, *Trachelyopterus* aff. *galeatus* from Araguaia River basin presented a chromosomal polymorphism in the same chromosomal pair (26) that was also reported to have a U2 snRNA chromosomal polymorphism [90]. Combining both polymorphic markers, U2 snRNA and SSR (GATA)_n_ resulted in three chromosomal forms. The B chromosomal form is exclusive of this population; whereas the C chromosomal form was also found in *T*. *galeatus* from Amazon River basin and the and A form is present in *T*. *striatulus*, *T*. *coriaceus*, *T*. *porosus*, and *Trachelyopterus* aff. *coriaceus*. Although both polymorphic states interact to compose the chromosome arrangement in *Trachelyopterus* aff. *galeatus*, they seem to be originated in different evolutive events. The U2 snRNA polymorphism is an exclusive trait of *Trachelyopterus* aff. *galeatus* and product of a pericentric inversion [90]. On the other hand, the additional (GATA)_n_ block that characterizes the polymorphism in *Trachelyopterus* aff. *galeatus* is not an exclusive trait and can also be seen in *T*. *galeatus* from Amazon River. Thus, the most parsimonious hypothesis is that the (GATA)_n_ polymorphism in *Trachelyopterus* aff. *galeatus* might be originated through hybridization in secondary contact zones [117,118], which could be facilitated by the historical and geomorphological aspects of the Araguaia River floodplains, known by constant ichthyofaunistic exchange across surrounding hydrographic systems during the neotectonics reactivations in the Transbrasiliano Lineament during the formation of the Araguaia depression [119–121].

Interestingly, the U2 snRNA inversion polymorphism in *Trachelyopterus* aff. *galeatus* was reported in Hardy-Weinberg equilibrium [90], in which, the spread of the polymorphism could be associated to the neutrality of the rearrangement, since it suggests that there is no change in adaptative value among the genotypes or in the host fitness [122], as reported for water beetles [123] and blackflies [124]. In this state, the polymorphism is essentially influenced by genetic drift and migration [122]. However, the polymorphic state of U2 snRNA with the (GATA)_n_ sequence, resulted in Hardy-Weinberg disequilibrium (Table 2), suggesting that the combined arrangement may be under the effect of different forces beyond just genetic drift and gene flow.

Furthermore, all genotypes were found in a similar proportion compared to the expected by the Hardy-Weinberg equilibrium test (Table 2), except the ones with the C chromosomal form, in which, none of the heterozygous were found in the sample (AC and BC) and the homozygous (CC) presented a three times higher frequency than expected. In some cases, heterozygous originated from chromosome rearrangements can suffer severe reductions in fitness [125], zygotic lethality [126] or hybrid [127,128], especially when it involves change in gene order within a chromosome (inversions) [125] or when the hybrids carry multiple rearrangements [125,126]. In this scenario, the presence of multiple chromosomal polymorphism in the same chromosomal pair, which origin of both could be related to major chromosome rearrangements (inversions), associated to the absence of the C heterozygous and higher frequency of the C homozygous, may suggest the existence of distinct evolutive pressures over it compared to other genotypes. However, analyzes with a larger sample size, since the C form in heterozygosis may just not have been collected, are still needed to clarify it.

In contrast to the previously discussed markers, the (GATA)_n_ chromosomal mapping on the 5S rDNA and H3/H4 histone gene carriers did not reveal new information about the structure of these chromosomal pairs. However, using the (GATA)_n_ distribution pattern on the 5S rDNA carriers, the possible chromosome homeologies between the species could be inferred (Fig 3). Since the 5S rDNA is a marker usually found on multiple chromosomal pairs in *Trachelyopterus*, the chromosomal correspondence is difficult to suggest without additional information about the organization of each chromosome, a gap that could be partially filled with the distribution of the (GATA)_n_ sequence on these chromosomes. Through the (GATA)_n_ mapping on the 5S rDNA chromosome pairs, three main chromosomal arrangements could be evidenced among the species (Fig 3), in which, the chromosomal arrangement (1) is present in all species, whereas the chromosomal arrangement (2) is present in *T*. *striatulus*, *T*. *galeatus*, *Trachelyopterus* aff. *coriaceus* and *T*. *coriaceus*, and the chromosomal arrangement (3) is present only in *T*. *striatulus* and *T*. *porosus*.

The microsatellite mapping combined with the H3 and H4 histone genes confirmed the distribution of SSR (GATA)_n_ already pointed out through the integration with 18S rDNA for the species that have this synteny (*T*. *striatulus*, *T*. *galeatus*, *Trachelyopterus* aff. *galeatus*, *T*. *porosus* and *T*. *coriaceus*) as well as the polymorphism in the chromosomal pair 20 of *T*. *galeatus* from the Amazon River basin. No new arrangement could be observed, and even for species that have multiple sites of H3 and H4 histone genes (*T*. *striatulus*, *T*. *galeatus*, *Trachelyopterus* aff. *galeatus* and *T*. *porosus*), both chromosomal pairs showed the same microsatellite distribution pattern.

### Integrated mapping perspectives to scattered microsatellites in Neotropical fishes

To date, there is no similar approach in cytogenetic mapping of microsatellites comparing Neotropical fish species. Most studies have focused in the origin and evolution of specific chromosomes, mainly B chromosomes (13 out of 81 studied species—16,04%) and sex chromosomes (24 out of 81 studied species– 29,62%); whereas others focused only on the type of array and presence or absence of the marker (Table 1). Nonetheless, of all cytogenetically analyzed species through physical mapping of microsatellites, 76.14% had at least one scattered microsatellite (67 out of 88 analyzed species), and in most studies, they could not be used to differ the species or populations.

Although the integrated mapping did not reveal large chromosomal rearrangements and most species had a similar distribution pattern of the (GATA)_n_ sequence in the analyzed chromosomes, it proved that the scattered microsatellite (GATA)_n_ has a non-random distribution, reiterating the existence of organization even in scattered microsatellites, which can be better described through a smaller scale analysis, comparing specific chromosomes between species. Through the integrated mapping a more accurate (GATA)_n_ pattern were described to the chromosome carriers of the 18S and 5S rDNA, H3 and H4 histone genes and U2 snRNA, and even though three species/populations used in this study were already mapped with (GATA)_n_, none of them were able to detect the (GATA)_n_ polymorphism in *T*. *galeatus* from Amazonas River basin and the presence of three chromosomal arrangements of the (GATA)_n_ sequence in the 5S rDNA carrier. Likewise, the (GATA)_n_ polymorphism in the U2 snRNA chromosomes of *Trachelyopterus* aff. *galeatus* from the Araguaia River basin would possibly go unnoticed without using the U2 snRNA site as a guide. Therefore, the integrated mapping (guided) proved to be an efficient methodology to reveal cryptic chromosomal arrangements of scattered microsatellites.

Cytotaxonomically, the integrated mapping showed the divergence in one more marker between *T*. *galeatus* from the Amazon River basin and *Trachelyopterus* aff. *galeatus* from the Araguaia River, which reiterate the existence of a possible new species, as already proposed through ribosomal markers [89] and integrated mapping with H3/H4 histone genes and U2 snRNA [90]. Methodologically, the integrated mapping turned the widely distributed SSR (GATA)_n_, with contributions in *Trachelyopterus* only to B chromosomes origin and evolution, into promising marker to distinguish other Auchenipteridae species. Thus, with the advancement of repetitive elements cytogenetic, we expect that the integrated mapping can further add to *Trachelyopterus* and also to other species with scattered distributed microsatellites.
